# The effects of saturated fatty acid supplements on plasma and milk concentration of fatty acid esters of hydroxy fatty acids in dairy cows

**DOI:** 10.3168/jdsc.2025-0813

**Published:** 2025-09-10

**Authors:** M. Arif, B.A. Harsch, C. Matamoros, I.J. Salfer, R. Shepardson, K.J. Harvatine

**Affiliations:** 1 Department of Animal Science, The Pennsylvania State University, University Park, PA 16802; 2 Department of Nutritional Sciences, The Pennsylvania State University, PA 16802; 3 Department of Animal Science, University of Minnesota, St. Paul, MN 55108

## Abstract

Fatty acid esters of hydroxy fatty acids (FAHFA) are a novel class of bioactive lipids with demonstrated antidiabetic and anti-inflammatory properties in rodent models and humans but have not been investigated in cows. The major FAHFA are synthesized from palmitic (PA), stearic (SA), and oleic acid. The PA can be esterified to hydroxy fatty acids, such as hydroxy PA or hydroxy SA, or SA can be esterified to hydroxy SA, forming PAHPA, PAHSA, or SAHSA, respectively. The objective of the current study was to determine the effect of increasing intake of PA, SA, or both PA and SA on nonesterified FAHFA in the plasma and milk of dairy cows. We hypothesized that increasing PA and SA in the diet would increase PA and SA containing FAHFA in plasma and milk. Samples were analyzed from a previous experiment that used 12 multiparous Holstein cows in a 4 × 4 Latin square design. Treatments were a no-fat supplement control (CON) and fat supplements that were high in PA (91% C16:0), high in SA (92.6% C18:0), or contained a blend of PA and SA (PA/SA; 45.3% C16:0 and 49.1% C18:0) at 1.95% of diet DM. The concentrations of nonesterified FAHFA in plasma and milk fat were quantified using liquid chromatography tandem MS, and data were analyzed using a mixed model that included treatment as a fixed effect and cow and period as random effects. The relationship between plasma FAHFA and milk production variables were analyzed using regression analysis. Five nonesterified FAHFA (9-PAHPA, 5-PAHSA, 9-PAHSA, 10-PAHSA, and 9-SAHSA) were quantified in plasma and all were affected by treatment. Plasma concentration of 9-PAHPA was increased 2.9-fold by PA compared with CON, whereas 9-SAHSA was increased 2.7-fold by SA compared with CON. The concentrations of 5-PAHSA, 9-PAHSA, and 10-PAHSA were highest with PA/SA. In milk, 8 nonesterified FAHFA were quantified, and only 12-PAHSA was increased by SA and 12-PAHPA tended to be increased by PA. Plasma 9-PAHPA was positively associated with milk fat yield and mixed FA and negatively associated with milk preformed FA, de novo FA, and odd- and branched-chain fatty acids, whereas plasma 9-SAHSA was positively associated with milk preformed FA. Overall, FA supplements affected nonesterified FAHFA concentration in plasma, demonstrating a direct effect of dietary FA on this emerging regulator of metabolism. There were limited effects of FA supplements on nonesterified FAHFA in milk fat. Functional roles for these lipids require further exploration.

## Highlights

• FAHFA are novel lipids associated with insulin resistance in rodents and humans.
• The effects of dietary PA and SA on FAHFA have not been explored in cows.
• Dietary PA and SA modified plasma FAHFA but had a limited impact on milk.

## Graphical Abstract

**Figure:**
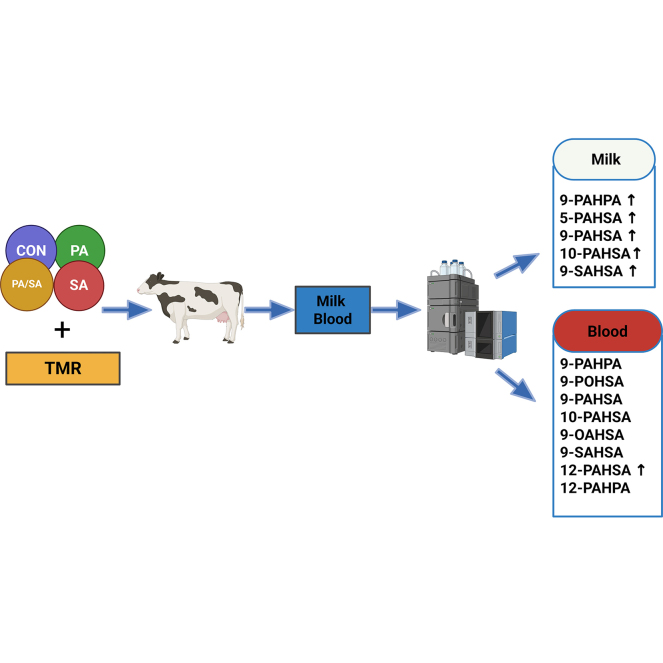
**Summary:** Fatty acid esters of hydroxy fatty acids (FAHFA) are synthesized by the esterification of fatty acids (FA) with hydroxy FA and have antidiabetic and anti-inflammatory properties in mice and humans. We explored the effects of dietary saturated FA supplementation on FAHFA concentration in plasma and milk. The supplementations of palmitic (PA) and stearic acids (SA) increased the FAHFA that are made by these FA and their hydroxy FA. However, there were limited effects of dietary supplementation on FAHFA in milk fat. Plasma FAHFA are responsive to dietary FA profile in dairy cows, and investigation of their synthesis and impact on metabolism requires further attention. Created in BioRender.

The fatty acid (**FA**) esters of hydroxy fatty acids (**HFA**), or **FAHFA**, are a novel class of bioactive lipids that consist of a FA esterified to a HFA. For example, palmitic acid (**PA**) can be esterified to HFA, such as hydroxy PA (**HPA**) or hydroxy stearic acid (**HSA**), forming **PAHPA** or **PAHSA**, respectively. The FAHFA can be categorized into families based on the FA and hydroxy FA they contain (e.g., PAHSA and PAHPA). They also differ within families based on the position of the estolide bond (e.g., 7-PAHSA vs. 9-PAHSA). The combination of different FA, HFA, and estolide position creates a large number of compounds, and the individual bioactivity appears to be regioisomer-specific (Yore et al., 2014).

The FAHFA have been observed in some plant (Liberati-Čizmek et al., 2019) and mammalian tissues (Yore et al., 2014). Specifically, they have been reported in plasma, adipose tissue, and milk of rodents and humans, as reviewed by Riecan et al. (2022), and their distribution is tissue-specific (Yore et al., 2014). Kellerer et al. (2021) reported that the serum FAHFA concentrations differed between omnivores versus vegetarians, and between obese people with or without diabetes. The FAHFA can be endogenously synthesized from absorbed or de novo synthesized FA and HFA. For example, Yore et al. (2014) observed that feeding 9-hydroxy heptadecanoic acid (15:0) increased tissue 9-palmitic acid-hydroxy heptadecanoic acid in mice. The FAHFA are found in the free form but also can be esterified in triglycerides (**TG**) and are stored in adipose tissue. In many tissues the esterified pool appears to be larger, but hormone-sensitive lipase (**HSL**; Human Genome Organization [HUGO]: LIPE) and adipose triglyceride lipase (**ATGL**; HUGO: PNPLA2) mobilize esterified FAHFA (Brejchova et al., 2021), and the physiological role of each form is not clear.

The FAHFA have been proposed to have beneficial effects in human and mouse models. Increases in the PAHSA family are associated with improving insulin sensitivity in mice (Zhou et al., 2019). The molecular mechanism is still under study, but the bioactive effect of 5- and 9-PAHSA appears to be mediated through the G-protein coupled receptors. It has been shown that the activation of GPR120 by 5- and 9-PAHSA increases insulin-stimulated translocation of GLUT4 in white adipose tissue, suggesting hypoglycemic and antidiabetic properties (Yore et al., 2014). Additionally, Yore et al. (2014) also observed that PAHSA enhances glucose-stimulated insulin secretion in mice, either by directly affecting pancreatic β cells or indirectly through the secretion of GLP-1. Additionally, PAHSA has been shown to protect against colitis by regulating innate and adaptive immune responses in mice (Lee et al., 2016).

To the best of our knowledge, FAHFA have not been characterized in dairy cows. Feeding PA and stearic acid (**SA**) is a common nutritional strategy in dairy cows, making them an interesting model to characterize the potential to modify FAHFA that may be responsible for some of the metabolic effects of FA supplements. The objectives of this study were to characterize the concentrations of free FAHFA in plasma and milk of dairy cows and to determine the effect of FA supplements. We hypothesized that diets supplemented with PA and SA would increase the plasma and milk concentrations of FAHFA that are synthesized by these FA and their HFA.

Samples were used from an experiment previously conducted in a tiestall barn at the Pennsylvania State University Dairy Research and Teaching Center (Shepardson and Harvatine, 2021). All animal procedures were approved by the Institutional Animal Care and Use Committee. Briefly, 12 multiparous lactating Holstein cows (138.5 ± 21.0 DIM, 53.4 ± 8.7 milk yield kg/d; mean ± SD) were arranged in a 4 × 4 Latin square design with 21-d experimental period. Treatments were a basal diet without supplemental fat (**CON**; 16.7% CP, 29.7% NDF, 17.7% ADF, 32.7% starch, 4.8% ash, and 3.26% FA on a DM basis) or the CON diet supplemented with an enriched 16:0 supplement (**PA**; 91.0% of FA), enriched 18:0 supplement (**SA**; 92.6% of FA), or a supplement containing a mixture of 16:0 and 18:0 (**PA/SA**; 45.3% 16:0 and 49.1% 18:0). All fat supplements were fed at 1.95% of diet DM.

Blood samples were taken 8 times on the last 3 d of each experimental period to represent every 3 h over the day from the coccygeal vein using potassium EDTA-coated Vacutainers (Griner Bio-One North America Inc.). After collection, samples were immediately centrifuged for 15 min at 1,300 × *g* at 4°C, and plasma was stored at −80°C for FAHFA analysis.

Nonesterified FAHFA were extracted as previously described (Zhang et al., 2016; Kolar et al., 2018) with modification. Liquid-liquid extraction was of lipids was followed by solid phase extraction to fractionate the FAHFA from other lipids. Briefly, 150 µL of plasma was mixed with 350 µL of citric acid buffer (1 *M* sodium chloride, 100 m*M* sodium citrate tribasic dihydrate, pH 3.6), 500 µL of methanol, 1 mL of chloroform, and 20 µL of ^13^C_16_-9-PAHSA as an internal standard. The mixture was vortexed for 30 s and centrifuged at 1,800 × *g* for 6 min at 4°C. The organic phase was transferred, dried with N_2_, and reconstituted in 200 µL of chloroform. A 3-mL SPE column (Sl-1 Silica 55 µm, 70 A, 500 mg/3 mL, Phenomenex) was prewashed with 3 mL of ethyl acetate and conditioned with 3 mL of hexane. Samples were added, and neutral lipids were washed with 3 mL of hexane:ethyl acetate (95:5) solution. The FAHFA fraction was eluted with 1 mL of ethyl acetate, dried under N_2_, and reconstituted with 100 µL of methanol containing 10 µ*M* 1-cyclohexyluriedo-3-dodecanoic acid (**CUDA**).

The FAHFA were quantified by liquid chromatography tandem MS using a Waters Acquity UPLC coupled to Waters Xevo triple quadrupole MS equipped with an electrospray ionization source (Waters, Milford, MA). Briefly, 5 µL of the extract was injected onto an Acquity UPLC BEH C18 column (2.1 × 50 mm with a 1.7 µ*M* particle size column). Flow rate was 750 µL/min using a gradient of water:acetonitrile (70:30) with 0.1% acetic acid (solvent A) and acetonitrile:isopropanol (50:50; solvent B) for 10 min (0 to 1.5 min from 0% B to 80% B, 1.5 to 8 min 85% B, 8 to 8.5 min 100% B, and 8.5 to 10 min 0% B). Electrospray ionization was operated in negative ion mode with the capillary set at 1.8 kV; desolvation and the source were 200°C and 150°C, respectively. The FAHFA multiple reaction monitoring transitions were found through direct injection of pure standards onto the mass spectrometer using cone voltage and collision energy ramps to optimize detection and the most prevalent daughter fragments. Calibration curves were generated before each run using standards for each FAHFA. Peak detection and integrations were done with Target Lynx (Waters, Milford, MA) and each peak was inspected for accuracy.

Data were analyzed in JMP PRO 17.0 (SAS Institute Inc.) with a model that included the random effects of period (1 to 4) and cow (n = 12) and the fixed effect of treatment (CON, PA, SA, and PA/SA). Studentized residuals outside of ±3 were considered outliers and excluded from analysis. Significance was declared at *P* < 0.05 and tendencies at *P* < 0.10. Means were separated using a protected LSD test.

Five nonesterified FAHFA were identified in plasma, including 9-PAHPA, 5-PAHSA, 9-PAHSA, 10-PAHSA, and 9-stearic acid-hydroxy stearic acid (**9-SAHSA**), and all were affected by treatment (*P* < 0.05, Figure 1). The concentration of 9-PAHPA was increased 2.9-fold by PA and 1.9-fold by PA/SA as compared with CON. Increasing PA had no effect on 9-SAHSA, but it was increased 2.7-fold by SA and 1.1-fold by PA/SA. Furthermore, PA/SA increased 5-PAHSA (8.8-fold), 9-PAHSA (4.7-fold), and 10-PAHSA (4.2-fold) as compared with CON. Increasing PA or SA alone resulted in similar increases in 5-PAHSA, 9-PAHSA, and 10-PAHSA as compared with CON. The greatest increases were observed in 5-PAHSA, with 4.1-, 3.5-, and 8.8-fold increases in PA, SA, and PA/SA, respectively. The 5 FAHFA identified in plasma were derived from PA and SA and those containing oleic acid were not detectable. The increase in plasma FAHFA in the current study is in agreement with Kellerer et al. (2021) who reported that increasing dietary SFA increased total FAHFA in humans. Kuda et al. (2016) observed that n-3 PUFA supplementation in humans and mice increased 9- and 13-docosahexaenoic acid hydroxyoctadecadienoic acid (9,13-DHAHLA) and 14-docosahexaenoic acid hydroxy docosahexaenoic acid (14-DHAHDHA), but plasma PAHSA levels remained unaltered. This suggests the dietary intake of a FA increases their corresponding FAHFA family in plasma and tissues. Saturated FA absorption is higher in ruminants than nonruminants as the rumen extensively biohydrogenates UFA, converting them to SFA, and SFA supplements are commonly fed to dairy cows to increase energy intake and milk fat yield without disrupting rumen fermentation.

**Figure 1:**
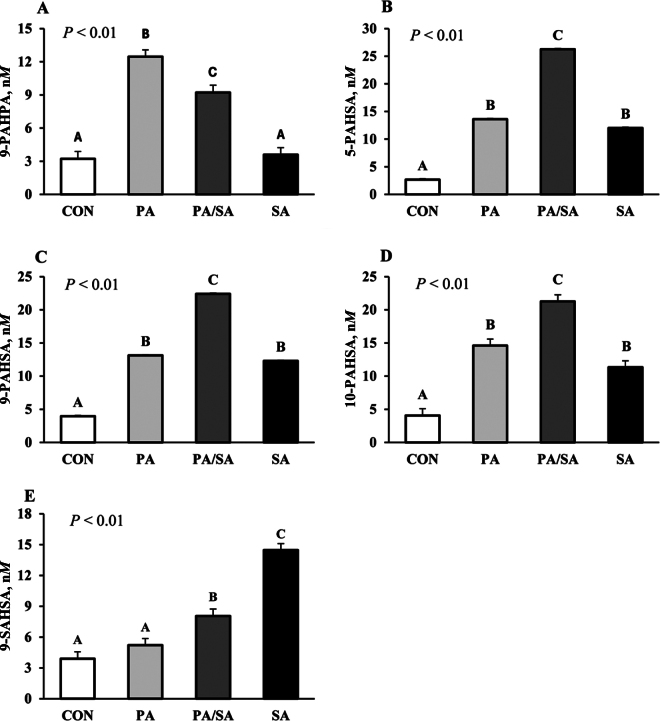
Effects of palmitic and stearic fatty acid supplements on plasma fatty acid hydroxy fatty acids (FAHFA). Treatments were a basal diet with no fat supplement (CON) or a high palmitic (PA; 91.0% C16:0), high stearic (SA; 92.6% C18:0), or a palmitic and stearic acid blend (PA/SA; 45.3% C16:0 and 49.1% C18:0) fed at 1.95% of diet DM. Data are presented as means ± SEM. Means sharing a letter do not differ (*P* < 0.05).

The current study quantified nonesterified FAHFA, but they are also incorporated into TG, which are referred to as FAHFA-TG. Esterified FAHFA have been investigated in adipose tissue (Tan et al., 2019), where their release from TG depends on HSL and ATGL (Brejchova et al., 2021). Several hydrolases, including CEL, ADTRP, AIG1, and HSL are potential FAHFA hydrolases as reviewed by Riecan et al. (2022). The rate of hydrolysis by CEL depends upon the position of ester bond and FA saturation, as unsaturated FAHFA with the ester bond further away from the carboxylate have a higher rate of hydrolysis. Hence, FAHFA esterification to TG or release from TG and their degradation to FA and HFA by hydrolases can also play an essential role in regulating their plasma levels.

The origin of the FAHFA found in plasma is not determinable in the current work. Dietary FAHFA have been reported in some foods including vegetables and cereals (Yore et al., 2014), but content in feedstuffs fed to dairy cows is not reported and ruminal metabolism of dietary FAHFA has also not been investigated to our knowledge. They are known to be synthesized in adipose or liver and are liberated from adipose TG during lipid mobilization. Although FAHFA synthesis in the gut has not been characterized, the ATGL enzyme is expressed in the gut (Obrowsky et al., 2013) and ATGL transacetylation has been reported to be capable of FAHFA synthesis (Patel et al., 2022). Increased FAHFA have been associated with lipid mobilization (Tan et al., 2019) and PA has been shown to increase BW loss in early lactation (de Souza and Lock, 2019). However, the cows in the current experiment were postpeak and expected to be in a positive energy balance. Additionally, the changes in plasma FAHFA mirrored the changes in FA profile of the dietary supplements that were fed for 21 d, indicating that they were likely from synthesis rather than longer-term storage.

Endogenous synthesis of FAHFA depends on the supply of both the FA and HFA and likely regulation of synthesis pathways. Palmitic acid is synthesized in adipose tissue through de novo lipogenesis and SA through elongation of PA. Both are also available from absorption, especially SA, which is the major FA available due to extensive rumen biohydrogenation. Thus, both PA and SA are abundantly available, and their supplementation clearly increased respective FAHFA in the current experiment indicating either a substrate-driven mechanism or changes in hydroxy FA or FAHFA synthesis pathways.

Hydroxy FA can be formed in the rumen through microbial metabolism of oleic acid (Jenkins et al., 2006), although synthesis of FAHFA in the rumen have not been specifically investigated. The treatments in the current experiment differed in saturated but not unsaturated FA, so synthesis in the reducing environment of the rumen is not expected. Hydroxy FA can also be endogenously synthesized in tissues by oxidizing saturated FA in phospholipids through a peroxireoxin-6 (PRDX6)-dependent pathway (Benlebna et al., 2021). The increase in HPA with PA feeding and HSA with SA feeding may be due to increased incorporation of these FA into phospholipids, although the regulation of these enzymes is not well understood.

The esterification of HFA with a FA to synthesize FAHFA has been reported to be catalyzed by ATGL (Patel et al., 2022). Regulation FAHFA synthesis is not well described, but is clearly responsive to PA and SA in the current experiment. Synthesized FAHFA can be released into plasma from tissues or esterified into TG and then subsequently liberated by hydrolysis by ATGL and HSL (Brejchova et al., 2021). The activity of these processes is not described in general nor for the bovine or mammary gland specifically. Free FAHFA can also be degraded to HFA and FA by numerous enzymes (CEL, ADTRP, and AIG1; Riecan et al., 2022).

A second hypothesis was that FA supplements would increase the concentration of nonesterified FAHFA in milk fat. Milk samples were collected from both milkings (0700 and 1800 h) on d 21 of each experimental period and composited by milk yield at each milking. Milk fat cakes were extracted by centrifugation for 20 min at 1,300 × *g* at 4°C. The nonesterified FAHFA concentration was analyzed with slight modification to the above. Briefly, ∼10 mg of fat cake, 1.5 mL of citric acid buffer, 1.5 mL of methanol, and 3 mL of chloroform were used for extraction and dried samples were reconstituted in 300 µL of chloroform. Additionally, 300 µL of 1:1 methanol:acetonitrile with 10 µ*M* CUDA was used as an internal standard. Data were analyzed as described previously.

Increasing dietary SA increased nonesterified 12-PAHSA in milk fat (0.95-fold, *P* < 0.01) and increasing PA tended to increase 12-PAHPA compared with CON (*P* = 0.07). The other FAHFA detected, including 9-PAHPA, 9-POHSA, 9-PAHSA, 10-PAHSA, 9-OAHSA, and 9-SAHSA, were not affected by treatment (*P* > 0.05, Figure 2). It is interesting that nonesterified FAHFA in milk fat did not mirror the changes in plasma FAHFA, suggesting either plasma FAHFA are not taken up by mammary epithelial cells, FAHFA are degraded in mammary epithelial cells or in milk, or they were differentially esterified in TG and depleted in the nonesterified pool quantified in this study. It is also possible that plasma esterified FAHFA rather than free FAHFA are driving milk fat FAHFA concentration. A tissue-specific distribution of FAHFA classes and their isomer has been reported in mice (Yore et al., 2014). Briefly, 7 PAHSA isomers were observed in brown, subcutaneous, and perigonadal adipose tissue and 5 in serum but only 2 in liver of mice. Additionally, they also reported 9-PAHSA was more abundant in adipose tissue than liver, whereas 12/13-PAHSA was higher in the liver than subcutaneous and perigonadal adipose.

**Figure 2:**
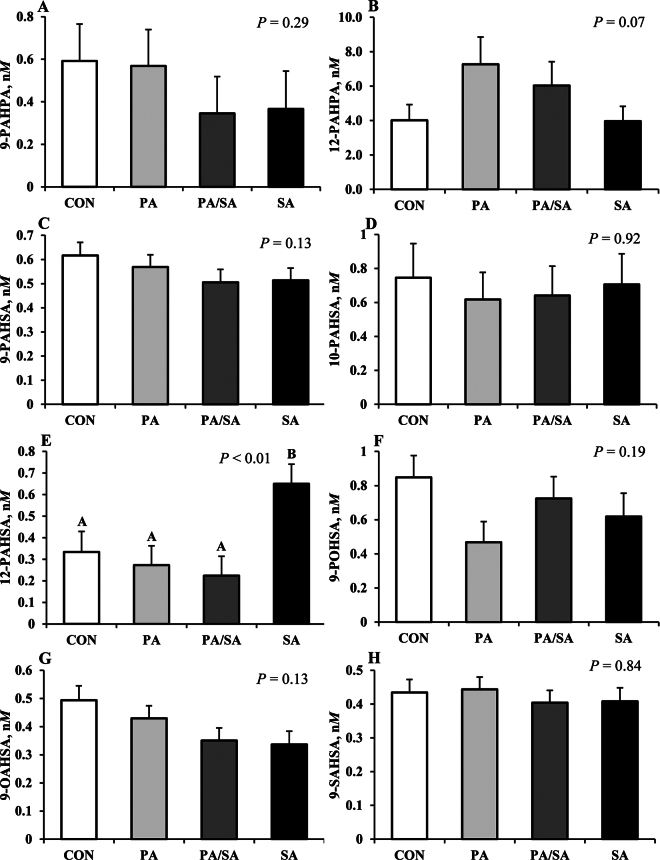
Effects of palmitic and stearic fatty acid supplements on milk fatty acid hydroxy fatty acids (FAHFA). Treatments were a basal diet with no fat supplement (CON) or a high palmitic (PA; 91.0% C16:0), high stearic (SA; 92.6% C18:0), or a palmitic and stearic acid blend (PA/SA; 45.3% C16:0 and 49.1% C18:0) fed at 1.95% of diet DM. Data are presented as means ± SEM. Means sharing a letter do not differ (*P* < 0.05).

Both unesterified FAHFA and FAHFA-TG have also been reported in human milk (Brejchova et al., 2022). Although difficult to directly compare, individual unesterified FAHFA were found at nanomolar concentrations, whereas TG containing FAHFA were found at micromolar concentrations. Brezinova et al. (2018) also observed an increase in total milk PAHSA concentrations in lactating human mothers who gained more BW during pregnancy, whereas 5-PAHSA and total PAHSA levels were higher in lean as compared with obese mothers. They proposed that FAHFA may originate from mammary adipose tissue or be transported to the mammary gland from circulation. In adipose tissue, esterified FAHFA were 100-fold greater than unesterified (Tan et al., 2019). Additional work is required to quantify the esterified FAHFA in cow milk fat.

We also explored the relationship between plasma FAHFA (independent variables) and milk production traits (dependent variables) by random regression. Briefly, the model included the random effect of cow and period and the fixed effect of plasma FAHFA concentration. The partial R^2^ for plasma FAHFA was calculated as the R^2^ of the regression after adjusting observations for the effect of cow and period.

Plasma 9-PAHPA was positively associated with milk fat yield (partial R^2^ = 0.27, *P* < 0.001) and mixed FA (partial R^2^ = 0.65, *P* < 0.001) and negatively associated with milk de novo FA (partial R^2^ = 0.59, *P* < 0.001), preformed FA (partial R^2^ = 0.39, *P* < 0.001), and odd- and branched-chain FA (partial R^2^ = 0.64, *P* < 0.001; Figure 3). Additionally, 9-SAHSA was positively related to milk preformed FA (partial R^2^ = 0.31, *P* < 0.001). These relationships are not unexpected based on the increase in PA-based FAHFA with PA feeding and the increase in milk fat yield through increased 16 C FA in milk fat. It is important to note that this is correlative, and mechanistic work that increases these FAHFA without changes in total PA and SA availability are needed. Plasma FAHFA were not related to milk or milk protein yield, and other FAHFA were not related to milk FA yield or concentration (data not shown).

**Figure 3:**
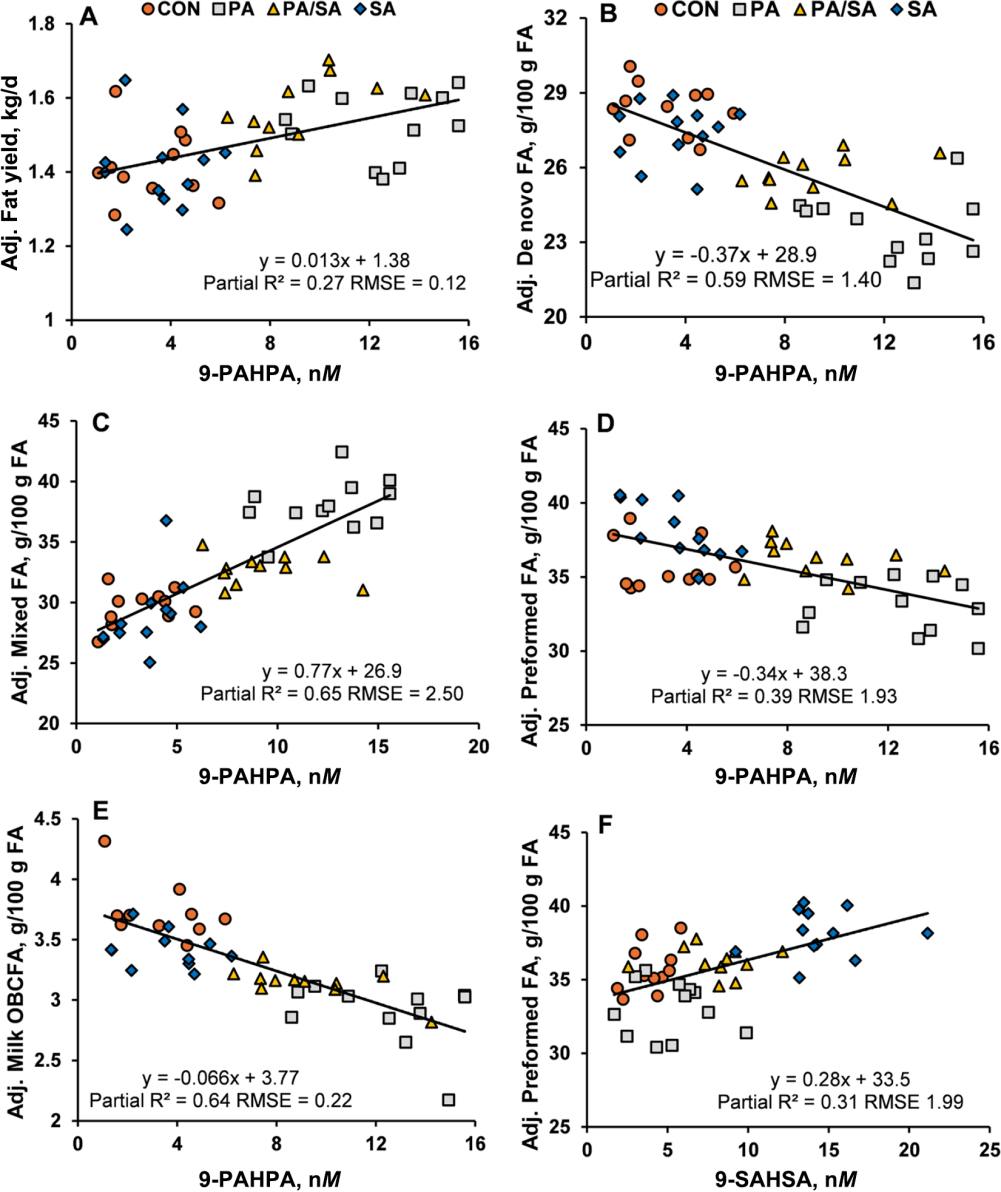
Significant relationships between milk traits and plasma FAHFA by random regression. Plotted data are adjusted (Adj.) to remove the random effects of cow and period. (A) Relationship between fat yield (kg/d) and 9-PAHPA (n*M*). (B) Relationship between de novo FA (g/100 g of FA) and 9-PAHPA (n*M*). (C) Relationship between mixed FA (g/100 g of FA) and 9-PAHPA (n*M*). (D) Relationship between preformed FA (g/100 g of FA) and 9-PAHPA (n*M*). (E) Relationship between OBCFA (g/100 g of FA) and 9-PAHPA (n*M*). (F) Relationship between preformed FA (g/100 g of FA) and 9-SAHSA (n*M*). Partial R^2^ of the effect of plasma FAHFA and root mean square error (RMSE) are shown in each panel. Regression was significant at *P* < 0.001 for all.

In conclusion, FAHFA concentrations in plasma were affected by PA and SA supplementation, but their concentrations in milk, with the exception of 12-PAHSA, were not affected. Our findings suggest that dietary FA supplements modulate plasma and milk FAHFA levels differently. Further studies are needed to investigate this differential response to FAHFA concentrations in plasma and milk to dietary FA supplementation. Moreover, future research could investigate the role of FAHFA in other aspects of lipid metabolism in dairy cows.
